# Inoculation of Transgenic Resistant Potato by *Phytophthora infestans* Affects Host Plant Choice of a Generalist Moth

**DOI:** 10.1371/journal.pone.0129815

**Published:** 2015-06-08

**Authors:** Kibrom B. Abreha, Erik Alexandersson, Jack H. Vossen, Peter Anderson, Erik Andreasson

**Affiliations:** 1 Department of Plant Protection Biology, Swedish University of Agricultural Sciences, Alnarp, Sweden; 2 Wageningen UR Plant Breeding, Wageningen University and Research Center, Wageningen, The Netherlands; Swedish University of Agricultural Sciences, SWEDEN

## Abstract

Pathogen attack and the plant’s response to this attack affect herbivore oviposition preference and larval performance. Introduction of major resistance genes against *Phytophthora infestans* (*Rpi*-genes), the cause of the devastating late blight disease, from wild Solanum species into potato changes the plant-pathogen interaction dynamics completely, but little is known about the effects on non-target organisms. Thus, we examined the effect of *P*. *infestans* itself and introduction of an *Rpi*-gene into the crop on host plant preference of the generalist insect herbivore, *Spodoptera littoralis* (Lepidoptera: Noctuidae). In two choice bioassays, *S*. *littoralis* preferred to oviposit on *P*. *infestans*-inoculated plants of both the susceptible potato (cv. Desiree) and an isogenic resistant clone (A01-22: cv. Desiree transformed with *Rpi-blb1*), when compared to uninoculated plants of the same genotype. Both cv. Desiree and clone A01-22 were equally preferred for oviposition by *S*. *littoralis* when uninoculated plants were used, while cv. Desiree received more eggs compared to the resistant clone when both were inoculated with the pathogen. No significant difference in larval and pupal weight was found between *S*. *littoralis* larvae reared on leaves of the susceptible potato plants inoculated or uninoculated with *P*. *infestans*. Thus, the herbivore’s host plant preference in this system was not directly associated with larval performance. The results indicate that the *Rpi-blb1* based resistance in itself does not influence insect behavior, but that herbivore oviposition preference is affected by a change in the plant-microbe interaction.

## Introduction

Plants are continuously exposed to pathogen attacks and damage by herbivores. As a result of the evolutionary arms race between pathogen and host, plants are equipped with defense signaling pathways [1, 2]. Induced response mechanisms after a pathogen attack as well as the effect of the infection itself change the plants’ transcriptome profile [3], phytohormone biosynthesis [4, 5], and chemical profile [6–8]. Herbivores use visual and/or chemical cues to locate and accept host plants, and pathogen infection is known to modulate herbivores’ host preference behavior, performance, and population dynamics and structure [9].

Pathogens cause considerable economic damage in plant production and introduction of major resistant (*R*) genes is considered as one of the most powerful option to reduce the effects by the pathogens [10]. However, little is known about the effect of *R* genes introduced into the plant against a target pathogen on herbivore host plant choice, if applied in agricultural fields. It is also important to estimate ecological consequences of the use of transgenic techniques to improve disease resistance in economically significant crops.

*Phytophthora infestans* is the causal agent of potato late blight, a disease with devastating economic impact. The pathogen caused the Irish potato famine in the 1840’s, and it poses a major threat to potato production causing estimated annual losses of billions of US dollars [11]. Many *R* genes against *P*. *infestans* have been transferred from wild *Solanum* species to potato [12, 13]. The *R* genes encode for *R* proteins, most of which are nucleotide-binding-leucine-rich-repeat (NB-LRR) type [14], which recognize avirulence effectors (Avr) of the pathogen, and initiate plant defense, restricting expansion of the infecting pathogen by inducing effector-triggered immunity (ETI). Inoculation of potato with *P*. *infestans* initiates a defense reaction changing the gene expression profile and abundance of secreted proteins [15–17], as well as volatile and non-volatile metabolite composition of the plant [6, 18]. However, studies investigating the effects of inoculation with *P*. *infestans* and introduction of late blight *R* genes on herbivores’ oviposition preference and larval performance are lacking. These types of studies are important in order to be able to predict consequences of the increased use of *R* genes in breeding that we foresee because of the increased availably of these genes and their markers.

The Egyptian cotton leafworm, *Spodoptera littoralis* (Boisduval) (Lepidoptera: Noctuidae), is a generalist insect herbivore with a wide range of host plant species, including potato [19]. Even though *S*. *littoralis* is a generalist, it shows selective responses to host plants [20] and to plant emitted volatiles during host plant choice [21, 22]. Induced changes in host plants after herbivore attack have been shown to have a large impact on host plant choice, and oviposition is reduced on plants damaged by feeding of conspecific larvae [23, 24] and by larvae of an underground herbivore [25].

According to the CABI invasive species compendium (http://www.cabi.org/isc/), *S*. *littoralis* is regarded as an invasive species that has been intercepted and quarantined in several countries. It is also a representative of opportunistic generalist insect species. Furthermore, expansion of areas with problems connected to *P*. *infestans* has also been reported [26]. In this study, we have investigated the effect of introduction of a classic *R* gene, *Rpi-blb1* from the *Solanum bulbocastanum*, against *P*. *infestans* on behavioral responses of the generalist insect herbivore *S*. *littoralis*. We hypothesized that inoculation of potato with *P*. *infestans* and introduction of the late blight *R* gene would affect host plant preference of *S*. *littoralis*. In addition, the relationship between the herbivore’s oviposition decision and its performance was tested. By using two-choice experiments, we show an increased oviposition preference of *S*. *littoralis* for susceptible and resistant isogenic potato plants inoculated with *P*. *infestans* compared with uninoculated plants. However, *P*. *infestans*-inoculated susceptible potato plants were preferred over inoculated resistant potato plants. The larvae performance test did not show a direct relationship with host preference.

## Materials and Methods

### Generation and growth of plant material

A genomic fragment of the *Solanum bulbocastanum Rpi-blb1* gene [27], including its native upstream and downstream regulatory elements, was introduced into the susceptible cv. Desiree using *Agrobacterium*-mediated transformation. Plants from the resulting transgenic clone A01-22 was analyzed in relation to expression of *Rpi-blb1* before inoculation, as well as 24, 48, 72 and 96 hours after inoculation by quantitative PCR in two biological replicates as described in Burra et al. [28].

Sterile *in vitro* plantlets were grown in a shooting-media (½MS (Murashige and Skoog) with vitamins, 20 g/L sucrose, 8 g/L agar, and 0.5 mg/L IBA (Indole-3-butyric acid), pH = 5.8) in controlled growth conditions with 16 h of light (80 μmol m^-2^s^-1^), day and night temperature of 23°C/18°C, and with approximately 65% relative humidity. Four week old plantlets were transferred from shooting media into plastic pots (1.5 L and ∅ = 15.1 cm) filled with soil. The transferred plantlets were covered with transparent plastic bags during the first week to avoid desiccation, and grown as described in Bengtsson et al. [29]. From the 8^th^ day, plantlets were watered regularly and supplied with a fertilizer solution (Rika S, Weibull Horto, Sweden). Six week old plants were used for both the host preference and larvae performance experiments.

### *P*. *infestans* maintenance and whole plant assay

A Swedish *P*. *infestans* strain SE-03058 (mating type A1) was used in this study [30]. A new batch of the pathogen was obtained every 3 months from cryopreserved stock, aseptically maintained on rye-A agar medium [31], incubated at 16°C with 16 h of light, and sub-cultured every second week. Sporangia were collected using milli-Q water from 12-day old *P*. *infestans* mycelium growing in a petri dish with rye-A agar media and filtered through a gamma irradiated cell strainer (∅ = 40 μm). The concentration of the spore suspension was adjusted to 15000 sporangia ml^-1^, kept at 4°C for 2 h, and shaken gently. Twenty μl of the spore suspension was drop inoculated on the abaxial side of detached leaves of the susceptible potato (cv. Desiree), placed in sealed transparent boxes with water moistened poly box sheet Katrin plus. The boxes were kept for 7–9 days at 16°C and 16 h of light, and spores released from the infected leaves as described in Moushib et al. [32] were used for whole plant inoculations of the host preference and larvae performance experiments.

Whole plant inoculation of six weeks old plants was carried out as indicated by Ali et al. [30], but plants were kept at 100% relative humidity for 24 hours after inoculation (hai).

### *S*. *littoralis* rearing

The *S*. *littoralis* were obtained from a laboratory colony, at SLU Alnarp in Sweden, founded on field-collected moths from Egypt in 2008. The colony has been refreshed with new wild-collected moths at least once a year in order to avoid inbreeding depression. The insects were maintained in rearing chamber at 25±2°C, 16 h light, and 65±5% relative humidity; and reared on a potato-based semi-synthetic diet [33]. Pupae were sorted by sex and males and females kept separate until the emerging adults were used in the experiments.

### Oviposition preference test

Oviposition preference of adult *S*. *littoralis* for either *P*. *infestans*-inoculated or uninoculated (control) plant of cv. Desiree and clone A01-22 was tested in net cages (BugDorm-44590 Insect Rearing Cage: 0.47 x 0.47 x 0.93 m) placed in the infection chamber set to 90% relative humidity. Four different sets of two-choice bioassays were carried out. Female oviposition preference was tested in the following four two-choice bioassays: 1) inoculated vs uninoculated cv. Desiree, 2) inoculated vs uninoculated clone A01-22, 3) cv. Desiree vs clone A01-22, both uninoculated, 4) cv. Desiree vs clone A01-22, both inoculated. While the first bioassay had three replicates, the other three were performed with two replicates, with 5–9 experimental cages in each replicate. At 24 hai, *P*. *infestans*-inoculated and uninoculated plants were positioned in opposite corners of an oviposition cage. A honeywater-saturated cotton ball in a plastic cup was placed in the middle of each oviposition cage as a supplementary food source for the insect. One female and two male adult *S*. *littoralis* were released into each cage. Eggs batches deposited within four days after the release of *S*. *littoralis* were collected and weighed separately.

### Larval performance test

For the larval performance test, *P*. *infestans*-inoculated and uninoculated plants of cv. Desiree were kept inside the infection chamber (90% relative humidity); leaves from the plants were detached at 24, 48, 72, or 96 hai to feed the larvae. First instars were individually transferred onto the detached potato leaves inside insect rearing boxes, using a fine brush. The larvae were distributed randomly into two treatment groups: inoculated and uninoculated cv. Desiree. Feeding on cotton leaves was included as a positive control because it is suitable host plant for *S*. *littoralis* [20]. To test if the pathogen could be a direct food source for the herbivore, *S*. *littoralis* larvae were also fed on *P*. *infestans* mycelium growing on agar plates. The experiments were performed at 25±2°C, 65±5% relative humidity, and 16 h light, with 10 larvae in each five boxes of each treatment group, 15–20 larvae in a box with cotton leaves, and 10–15 larvae in a plate with *P*. *infestans* mycelium. Each day, larvae were provided with sufficient and equivalent fresh detached leaves, the leftover leaves were removed, and boxes were thoroughly cleaned. Starting from day 3, the larvae were weighed every second day and treatment effects were determined based on the average weight at each weighing day. The experiment was repeated twice with the same setup and showed a consistent result.

### Statistical analysis

Oviposition preference data were analyzed separately for each two-choice bioassay; with treatment, replication, and cages as sources of variation. For the larvae performance test, the sources of variation were treatment and rearing box, and analyzed for each measurement day separately. Normality and homoscedasticity of the residuals were checked with graphics, and attested with Shapiro-Wilk normality test. Then the egg weight and larvae weight data, rounded to the nearest integer, were analyzed using a generalized linear mixed model (GLMM) in R-package ‘lme4’ v.1.1–7, using the glmer function with family poisson and nested random-effects terms, fit by maximum likelihood (Laplace approximation, nAGQ = 1)” [34]. Pairwise comparisons among the treatments were carried out using the R-package ‘multcomp’[35]; where p-values are adjusted using the single step method. All the statistical tests were conducted using a freely available statistical package ‘R v3.1.0’[36] in ‘RStudio v0.98.’ (http://www.rstudio.com). The significance level was set to α = 0.05.

## Results

### *Rpi-blb1* confers resistance against *P*. *infestans* strain SE-03058

**Fig 1:**
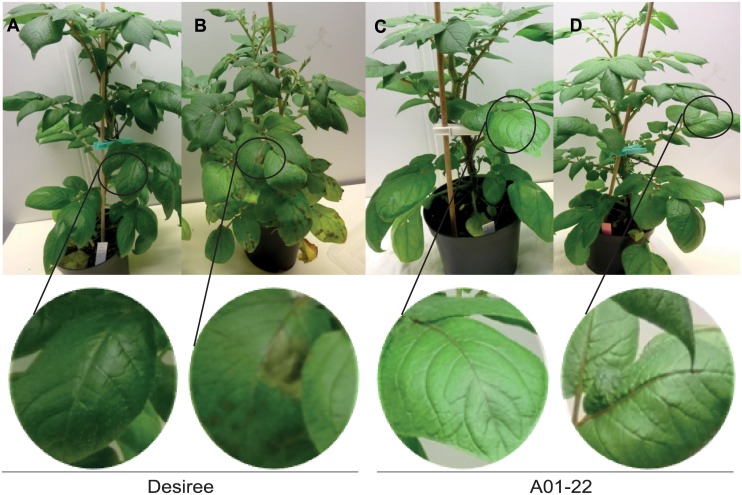
Whole plant infection of cv. Desiree and clone A01-22 (cv. Desiree transformed with *Rpi-blb1*) with *P*. *infestans*. Six week old plants of cv. Desiree and clone A01-22 were transferred to an infection chamber set to 100% relative humidity. After 6 h, plants were either left uninoculated (A & C) or inoculated with *P*. *infestans* (B & D). At 24 h after inoculation plants were placed in a net cage in the infection chamber that was set to 90% relative humidity. Pictures were taken at 5 days after inoculation.

At five days after inoculation, late blight symptoms were observed on the *P*. *infestans* inoculated cv. Desiree plants (Fig 1A and 1B). As expected, no symptoms on transgenic Desiree plants (clone A01-22), harbouring the *Rpi-blb1* gene, was observed at 5 dai (Fig 1C and 1D). Likewise, in a detached leaf assay, cv. Desiree showed lesion size of 375 mm^2^ (SD ± 40) at 7 dai, whilst the lesion size in A01-22 was restricted to the point of inoculation (data not shown). Thus, as shown with other strains of *P*. *infestans* and potato genotypes [27], the *Rpi-blb1* gene in clone A01-22 confers resistance against the strain (SE-03058). In this clone, mean expression of *Rpi-blb1* increases from 0.27+/-0.12 SD before inoculation to 1.04 +/- 0.09 SD at 48 hai for example (S1 Fig).

### *P*. *infestans* inoculation affects *S*. *littoralis* oviposition behavior

**Fig 2:**
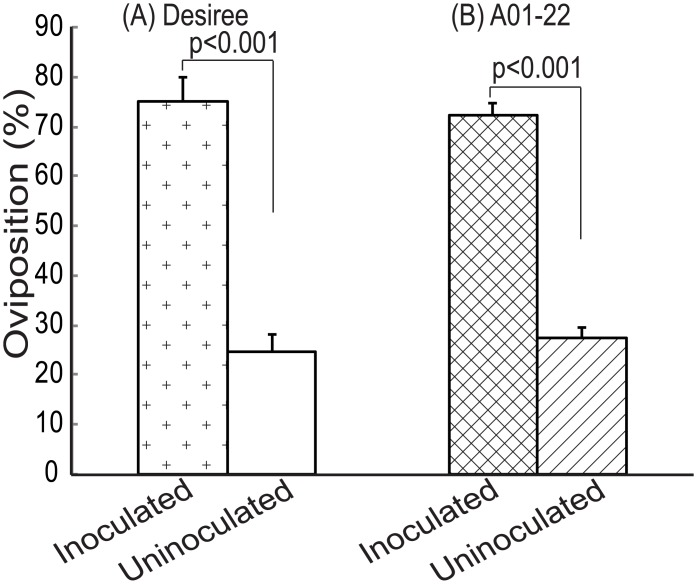
Two-choice test between *P*. *infestans* inoculated and uninoculated potato. *S*. *littoralis* oviposition preference between: (A) inoculated and uninoculated cv. Desiree, n = 15 paired plants (B) inoculated and uninoculated clone A01-22, n = 17 paired plants. At day four after release of insects, oviposited eggs were collected and weighed for each plant. Tukey pairwise comparisons was used for statistical analysis with significant difference set to α = 0.05. Bars indicate mean (±SE) percentage of eggs laid.

In a two-choice test between inoculated and uninoculated cv. Desiree (Fig 2A), a significantly higher proportion of *S*. *littoralis* eggs was laid on the *P*. *infestans* inoculated (75% ± 4.8 SE) than on the uninoculated plants (25% ± 2.2 SE) (Tukey pairwise comparison; *Z*_*15*_ = 9.2, *p* ±0.001). Similarly, 72% (± 3.2 SE) of the eggs were laid on *P*. *infestans* inoculated and 28% (± 2.3 SE) on the uninoculated plants of clone A01-22 (Tukey pairwise comparison; *Z*_*17*_ = 8.5, *p* ≤0.001) (Fig 2B). The above results show that *S*. *littoralis* prefers ovipositing on the *P*. *infestans* inoculated potato plants.

### *Rpi-blb1* alters host preference for oviposition only after inoculation

**Fig 3:**
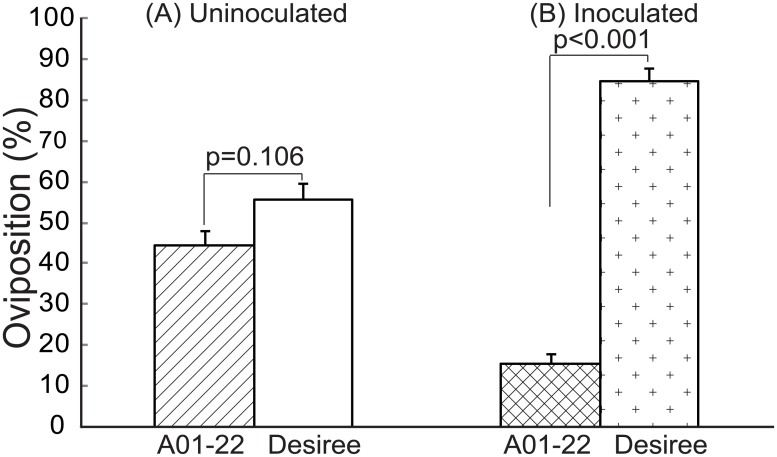
Two-choice test between cv. Desiree and clone A01-22 plants. *S*. *littoralis* was released into net cages to choose between (A) Uninoculated cv. Desiree and clone A01-22 plants, n = 11; and (B) *P*. *infestans* inoculated cv. Desiree and clone A01-22 plants, n = 13. Oviposited eggs within 4 days after the insect release were collected and weighed for each plant. Tukey pairwise comparisons was used for statistical analysis and significant difference set to α = 0.05. Bars indicate mean (±SE) percentage of eggs laid.

Two-choice tests, between cv. Desiree and clone A01-22, were performed in order to show the effect of introduction of *Rpi-blb1* on oviposition preference of *S*. *littoralis* on inoculated and uninoculated plants. When plants were not inoculated with the pathogen (Fig 3A), eggs were evenly distributed between the susceptible cv. Desiree (55% ± 4.1 SE) and resistant clone A01-22 (45% ± 3.4 SE) (Tukey pairwise comparison; *Z*_*11*_ = 1.6, *p* = 0.106). This result showed that the introduction of the *Rpi-blb1* gene into cv. Desiree itself did not affect oviposition. However, a higher percentage of *S*. *littoralis* eggs (Fig 3B) were laid on the *P*. *infestans* inoculated cv. Desiree (85% ± 3.3 SE) than on the inoculated clone A01-22 (15% ± 2.2 SE) (Tukey pairwise comparison; *Z*_*13*_ = 10.3, *p* ≤ 0.001). This change in distribution of eggs only after inoculation with *P*. *infestans*, suggests that the herbivore had a preference for diseased plants over healthy plants.

### Oviposition decision is not directly associated with larval performance

**Fig 4:**
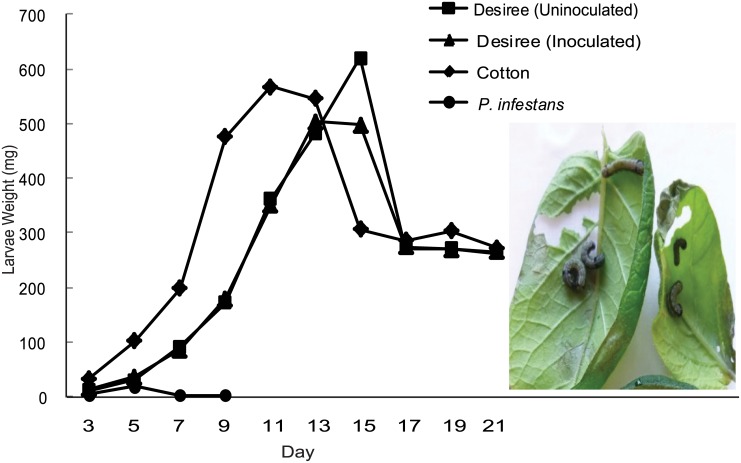
*S*. *littoralis* larvae performance test. First instar larvae of *S*. *littoralis* was fed on detached leaves of either *P*. *infestans* inoculated or uninoculated cv. Desiree, and Cotton or on *P*. *infestans* mycelium. Larvae were weighed every 48 hours. At day 17 all the larvae were transformed in to pupa. The experiment was repeated twice with similar results.

Larvae performance was compared on detached leaves from inoculated and uninoculated plants of cv. Desiree because it was on this genotype we detected the largest differences in oviposition between inoculated vs uninoculated plants (Fig 4). We performed GLMM tests to compare the effect of diet on larval weight for all four treatments (cotton, inoculated cv. Desiree, uninoculated cv. Desiree, and *in vitro* grown *P*. *infestans* mycelium) on days 3–9. Only on day 7 and day 9, larvae fed on *P*. *infestans* mycelium showed reduced weight compared to larvae on the other three diets (P<0.001). However, on day 9, the larvae fed on detached leaves of the positive control (cotton) were also significantly heavier than larvae fed on detached leaves form inoculated cv. Desiree (*p* ≤ 0.001) and uninoculated cv. Desiree (*p* ≤ 0.001).

The larvae placed inside petri dishes containing growing *P*. *infestans* mycelium on rye-A agar media increased in weight for the first two days, but afterwards showed a decreased weight and all had died on day 9 (Fig 4). Therefore, the three survival-treatments (cotton, inoculated cv. Desiree, uninoculated cv. Desiree) were compared on day 11–21, whereof day 17–21 data were on pupal weight. Larvae fed from the first instar until pupation on the detached leaves of either *P*. *infestans*-inoculated or uninoculated plants of the susceptible cv. Desiree and did not show significant difference in larval or pupal weight (*p* > 0.05), except on day 15 (*p* = 0.003). We also observed that feeding larvae tended to exclude the blighted part of inoculated leaves (Fig 4, right part), but later this was also consumed when the available leaf material was reduced (data not shown). We conclude that the host plant preference for oviposition of *S*. *littoralis* is not directly associated with the larvae performance.

## Discussion

This study shows that *P*. *infestans* resistance mediated by the *Rpi-blb1* gene in clone A01-22 has an effect on host preference behavior of a generalist insect herbivore. We demonstrate that *P*. *infestans* inoculation of both the susceptible cv. Desiree and the resistant clone A01-22 (cv. Desiree transformed with *Rpi-blb1*) had a significant effect on oviposition preference of adult *S*. *littoralis*. Furthermore, the *Rpi-blb1* of clone A1-22 affects *S*. *littoralis* oviposition behavior only when this *R* gene was activated by inoculation with the pathogen, because plants of the uninoculated cv. Desiree or clone A01-22 were equivalently preferred for oviposition by *S*. *littoralis*. However, in experiments using cv. Desiree and clone A01-22, when both were inoculated with the pathogen, significantly higher proportion of the eggs was deposited on the cv. Desiree plants.

The *Rpi-blb1* gene from *S*. *bulbocastanum* confers resistance against *P*. *infestans* [27, 37] including the pathogen strain used in this study. Major *R* gene-based resistance leads to the effector-triggered immunity (ETI), a layer of defense that susceptible plants lack [38]. Both cv. Desiree and clone A01-22 are genetically identical except for the *Rpi-blb1* gene. The expression of the *Rpi-blb1* gene in clone A01-22 increased 3–4 fold within 48 hai in the incompatible interaction compared to the uninoculated control. Therefore, the lower *S*. *littoralis* oviposition in the inoculated resistant clone A01-22 than on inoculated susceptible cv. Desiree could be attributed either to the activation of *Rpi-blb1*-mediated defence response or to the lower levels of *P*. *infestans* in the resistant plants.

Increased oviposition was found on plants inoculated with *P*. *infestans* in both cv. Desiree and clone A01-22. In accordance with this study, higher oviposition of herbivores on pathogen-infected host plants was previously reported in different pathosystems. In laboratory and greenhouse assays, Jallow et al. [39] found that the polyphagous moth *Helicoverpa armigera* (Hübner) deposited more eggs on tomato leaves inoculated with root fungal endophyte (*Acremonium strictum*) than on uninoculated leaves. Similarly, shoots of *Cirsium arvense* infected with rust fungus (*Puccinia punctiformis*) were more preferred for oviposition by a stem boring weevil (*Apion onopordi)* than uninfected shoots [40]. However, herbivores do not always prefer to oviposit on pathogen-inoculated host plants. For example, the beet armyworm *S*. *exigua* avoided oviposition on powdery mildewed (*Podosphaera pannosa*) leaves of *Rosa chinensis* [8], the leaf-feeding tortoise beetle *Cassida rubiginosa* preferred healthy over *Phoma destructiva*-inoculated plants of *Cirsium arvense* [41], and the leaf beetle *Phaedon cochleariae* avoided oviposition on chinese cabbage infected with *Alternaria brassicae* [42]. The lack of consensus among the results from our and previous studies suggests that currently no general conclusions can be made.

Our results indicate that the presence of *P*. *infestans* or induced responses after the infection affect the oviposition choice. During the larvae performance test, we observed an avoidance of the blighted parts of the leaves from *P*. *infestans* inoculated plants of cv. Desiree, and even death when feeding on the pathogen mycelium growing on rye-A agar. In addition, the feeding experiment on detached leaves from inoculated and uninoculated cv. Desiree leads to similar larvae performance. This indicates that *P*. *infestans* growing on the leaves of infected plants is not a food source for the larvae. As the inoculated resistant clone was preferred over than the uninoculated one, it may be the induced response in the plant to the infection of *P*. *infestans* that is most important for the ovipositing female rather than presence of the pathogen.

The results of this study differ to those found for damage induction by insect herbivores, where cotton plants attacked by conspecific larvae [23, 24] and by a below-ground herbivore [25] received fewer *S*. *littoralis* eggs than undamaged plants. Damage to a plant by herbivores and by pathogens affect the chemical profile of plants, inducing production of both volatile and non-volatile compounds [43]. It is shown that *P*. *infestans* inoculation changes the expression profile of genes also in resistant combinations [15–17] leading to accumulation of non-volatile phytoalexins, glycoalkaloids and phenolics [18], and alters the volatile fingerprints of potato plants [6]. There are normally differences in the induced chemical response in plants attacked by different plant enemies, such as pathogens and herbivores [43]. Such differences in the profile of volatile and non-volatile compounds could explain that *S*. *littoralis* females oviposit more on pathogen attacked plants, while they reduce oviposition on herbivore-induced plants.

Inoculation with *P*. *infestans* showed late blight disease development on the susceptible cv. Desiree but not A01-22. However, there was no significant weight difference between larvae of *S*. *littoralis* that fed on leaves of *P*. *infestans* inoculated and uninoculated plants of the susceptible cv. Desiree. In agreement with this result, infection of tomato leaf with *P*. *infestans* did not affect growth rates of *S*. *exigua* or the corn earworm *Helicoverpa zea* [44]. However, it has been reported that pathogen infection can affect nutritional quality of the plant [45]. In spite of similar amount of leaf consumption, the larvae of *S*. *littoralis* showed slightly higher weight after feeding on leaves of maize seedlings colonized by an endophytic bacterium (*Enterobacter aerogenes*) than on leaves of the healthy plants [46]. Larvae of *S*. *exigua* fed on white mold fungus (*Sclerotium rolfisii*) infected peanut plants [45] and on leaves of pepper plants infected with *Xanthomonas* [7] showed a better performance. Conversely, larvae of *S*. *exigua* fed on mildewed leaves showed decreased pupal weights, emergence rates, and fecundity [8]. Similarly, prolonged development time and decreased larval weights due to infection with fungi have been found in several species of beetles [41, 42, 47]. Our result does not show a link between female oviposition preference and larval performance. A recent meta-analysis [48] showed that there is often a mismatch between host preference and performance in generalist insect species.

It is also possible that use of detached leaves influenced the larval performance. In laboratory studies this is a common method used to test larval development or feeding efficiency [23, 41, 42, 49, 50]. Validating the larvae performance on an intact plant could be interesting. However, larval feeding would induce changes in host plant defense that could affect larval development, so the effects of the pathogen and the plant response to the pathogen could not be studied by themselves on intact plants.

One way to mitigate problems posed by *P*. *infestans* is to introduce *R* genes, and the availability of new types of *R* genes is constantly increasing. The *Rpi-blb1* shows a significant impact on late blight disease development in the field [51]. There are ecological concerns regarding the use of genetic engineering approaches to improve agronomic performance of crop plants, including direct effects on non-target organisms. Further studies with other transgenic *R* gene clones, other insect species, and large-scale field experiments are required to make a full conclusion. This study, however, suggests that a strategy of transgenic *R* gene introduction against potential pathogens may not increase the load of generalist insect herbivores, when plants are less infested by *P*. *infestans*.

## Supporting Information

S1 Fig Mean expression of the *Rpi-blb1* gene in the A01-22 clone. Expression anlaysis of the *Rpi-blb1* in clone A01-22 plants, at different time points after inoculation, was quantified using qPCR. Error bars indicate standard deviation of the expression in relation to the EF1 internal control.
(DOCX)

## References

[1] Pieterse CM, Dicke M. Plant interactions with microbes and insects: from molecular mechanisms to ecology. Trends in plant science. 2007;12(12):564–9. 10.1016/j.tplants.2007.09.004 . 17997347

[2] Hatcher PE, Moore J, Taylor JE, Tinney GW, Paul ND. PHYTOHORMONES AND PLANT–HERBIVORE–PATHOGEN INTERACTIONS: INTEGRATING THE MOLECULAR WITH THE ECOLOGICAL. Ecology. 2004;85(1):59–69. 10.1890/02-0724

[3] Rushton PJ, Somssich IE, Ringler P, Shen QXJ. WRKY transcription factors. Trends in plant science. 2010;15(5):247–58. 10.1016/j.tplants.2010.02.006 WOS:000278197500002. 20304701

[4] Blanco-Ulate B, Vincenti E, Powell AL, Cantu D. Tomato transcriptome and mutant analyses suggest a role for plant stress hormones in the interaction between fruit and Botrytis cinerea. Frontiers in plant science. 2013;4:142 10.3389/fpls.2013.00142 23717322 PMC3653111

[5] Robert-Seilaniantz A, Grant M, Jones JDG. Hormone Crosstalk in Plant Disease and Defense: More Than Just JASMONATE-SALICYLATE Antagonism. Annual Review of Phytopathology, Vol 49. 2011;49:317–43. 10.1146/annurev-phyto-073009-114447 WOS:000294828400016. 21663438

[6] Laothawornkitkul J, Jansen RMC, Smid HM, Bouwmeester HJ, Muller J, van Bruggen AHC. Volatile organic compounds as a diagnostic marker of late blight infected potato plants: A pilot study. Crop Protection. 2010;29(8):872–8. 10.1016/j.cropro.2010.03.003

[7] Cardoza Y, Tumlinson J. Compatible and Incompatible Xanthomonas Infections Differentially Affect Herbivore-Induced Volatile Emission by Pepper Plants. J Chem Ecol. 2006;32(8):1755–68. 10.1007/s10886-006-9107-y 16900430

[8] Yang FZ, Li Y, Yang B. The inhibitory effects of rose powdery mildew infection on the oviposition behaviour and performance of beet armyworms. Entomol Exp Appl. 2013;148(1):39–47. 10.1111/Eea.12069 WOS:000320724200004.

[9] Tack AJM, Dicke M. Plant pathogens structure arthropod communities across multiple spatial and temporal scales. Functional Ecology. 2013;27(3):633–45. WOS:000319420500007.

[10] Fry W. Phytophthora infestans: the plant (and R gene) destroyer. Molecular plant pathology. 2008;9(3):385–402. 10.1111/j.1364-3703.2007.00465.x . 18705878 PMC6640234

[11] Haverkort AJ, Struik PC, Visser RGF, Jacobsen E. Applied Biotechnology to Combat Late Blight in Potato Caused by Phytophthora Infestans. Potato Res. 2009;52(3):249–64. 10.1007/s11540-009-9136-3 WOS:000208413800007.

[12] Vleeshouwers VGAA, Raffaele S, Vossen JH, Champouret N, Oliva R, Segretin ME, et al Understanding and Exploiting Late Blight Resistance in the Age of Effectors. Annual Review of Phytopathology, Vol 49. 2011;49:507–31. 10.1146/annurev-phyto-072910-095326 WOS:000294828400024. 21663437

[13] Rodewald J, Trognitz B. Solanum resistance genes against Phytophthora infestans and their corresponding avirulence genes. Molecular plant pathology. 2013;14(7):740–57. 10.1111/Mpp.12036 WOS:000322591700008. 23710878 PMC6638693

[14] Zhang Y, Lubberstedt T, Xu ML. The Genetic and Molecular Basis of Plant Resistance to Pathogens. J Genet Genomics. 2013;40(1):23–35. WOS:000314116400003. 10.1016/j.jgg.2012.11.003 23357342

[15] Orłowska E, Fiil A, Kirk H-G, Llorente B, Cvitanich C. Differential gene induction in resistant and susceptible potato cultivars at early stages of infection by Phytophthora infestans. Plant Cell Rep. 2012;31(1):187–203. 10.1007/s00299-011-1155-2 21965005

[16] Ros B, Thummler F, Wenzel G. Comparative analysis of Phytophthora infestans induced gene expression in potato cultivars with different levels of resistance. Plant Biology. 2005;7(6):686–93. 10.1055/s-2005-872946 WOS:000234280600014. 16388472

[17] Ali A, Alexandersson E, Sandin M, Resjo S, Lenman M, Hedley P, et al Quantitative proteomics and transcriptomics of potato in response to Phytophthora infestans in compatible and incompatible interactions. BMC genomics. 2014;15(1):497 10.1186/1471-2164-15-497 . 24947944 PMC4079953

[18] Andreu A, Oliva C, Distel S, Daleo G. Production of phytoalexins, glycoalkaloids and phenolics in leaves and tubers of potato cultivars with different degrees of field resistance after infection with Phytophthora infestans. Potato Res. 2001;44(1):1–9. 10.1007/Bf02360281 WOS:000169640500001.

[19] Brown ES, Dewhurst CF. The genus spodoptera (Lepidoptera, Noctuidae) in Africa and the Near East. Bulletin of Entomological Research. 1975;65(02):221–62. 10.1017/S0007485300005939

[20] Thoming G, Larsson MC, Hansson BS, Anderson P. Comparison of plant preference hierarchies of male and female moths and the impact of larval rearing hosts. Ecology. 2013;94(8):1744–52. 10.1890/12-0907.1 WOS:000322336600009. 24015518

[21] Saveer AM, Kromann SH, Birgersson G, Bengtsson M, Lindblom T, Balkenius A, et al Floral to green: mating switches moth olfactory coding and preference. P Roy Soc B-Biol Sci. 2012;279(1737):2314–22. 10.1098/rspb.2011.2710 WOS:000303888500004. 22319127 PMC3350682

[22] Zakir A, Bengtsson M, Sadek MM, Hansson BS, Witzgall P, Anderson P. Specific response to herbivore-induced de novo synthesized plant volatiles provides reliable information for host plant selection in a moth. J Exp Biol. 2013;216(17):3257–63. 10.1242/Jeb.083188 WOS:000322955200020. 23737555

[23] Anderson P, Alborn H. Effects on oviposition behaviour and larval development of Spodoptera littoralis by herbivore-induced changes in cotton plants. Entomol Exp Appl. 1999;92(1):45–51. 10.1046/j.1570-7458.1999.00523.x WOS:000081687300006.

[24] Zakir A, Sadek MM, Bengtsson M, Hansson BS, Witzgall P, Anderson P. Herbivore-induced plant volatiles provide associational resistance against an ovipositing herbivore. Journal of Ecology. 2013;101(2):410–7. 10.1111/1365-2745.12041 WOS:000317923300015.

[25] Anderson P, Sadek MM, Wackers FL. Root herbivory affects oviposition and feeding behavior of a foliar herbivore. Behav Ecol. 2011;22(6):1272–7. 10.1093/beheco/arr124 WOS:000296295000026.

[26] Hannukkala AO, Kaukoranta T, Lehtinen A, Rahkonen A. Late-blight epidemics on potato in Finland, 1933–2002; increased and earlier occurrence of epidemics associated with climate change and lack of rotation. Plant Pathol. 2007;56(1):167–76. 10.1111/j.1365-3059.2006.01451.x WOS:000243507800020.

[27] van der Vossen E, Sikkema A, Hekkert B, Gros J, Stevens P, Muskens M, et al An ancient R gene from the wild potato species Solanum bulbocastanum confers broad-spectrum resistance to Phytophthora infestans in cultivated potato and tomato. The Plant journal: for cell and molecular biology. 2003;36(6):867–82. . 14675451 10.1046/j.1365-313x.2003.01934.x

[28] Burra DD, Berkowitz O, Hedley PE, Morris J, Resjo S, Levander F, et al Phosphite-induced changes of the transcriptome and secretome in Solanum tuberosum leading to resistance against Phytophthora infestans. BMC plant biology. 2014;14. WOS:000342782300001. 10.1186/s12870-014-0254-y PMC4192290 25270759

[29] Bengtsson T, Weighill D, Proux-Wera E, Levander F, Resjo S, Burra DD, et al Proteomics and transcriptomics of the BABA-induced resistance response in potato using a novel functional annotation approach. BMC genomics. 2014;15:315 10.1186/1471-2164-15-315 WOS:000335409900001. 24773703 PMC4234511

[30] Ali A, Moushib LI, Lenman M, Levander F, Olsson K, Carlson-Nilson U, et al Paranoid potato: Phytophthora-resistant genotype shows constitutively activated defense. Plant signaling & behavior. 2012;7(3):400–8. 22476463 10.4161/psb.19149 PMC3443922

[31] Caten CE, Jinks JL. Spontaneous variability of single isolates of Phytophthora infestans. I. Cultural variation. Can J Botany. 1968;46(4):329–48. 10.1139/b68-055

[32] Moushib L, Witzell J, Lenman M, Liljeroth E, Andreasson E. Sugar beet extract induces defence against Phytophthora infestans in potato plants. Eur J Plant Pathol. 2013;136(2):261–71. 10.1007/s10658-012-0160-9

[33] Hinks CF, Byers JR. BIOSYSTEMATICS OF THE GENUS EUXOA (LEPIDOPTERA: NOCTUIDAE): V. REARING PROCEDURES, AND LIFE CYCLES OF 36 SPECIES. The Canadian Entomologist. 1976;108(12):1345–57. 10.4039/Ent1081345-12

[34] Bates D, Maechler M, Bolker B, Walker S. lme4: Linear mixed-effects models using Eigen and S4. R package version 1.1–6. http://CRANR-projectorg/package=lme4. 2014.

[35] Hothorn T, Bretz F, Westfall P. Simultaneous Inference in General Parametric Models. Biometrical Journal. 2008;50(3):346–63. 10.1002/bimj.200810425 18481363

[36] R Core Team. R: A language and environment for statistical computing. R Foundation for Statistical Computing, Vienna, Austria. URL http://www.R-projectorg/. 2014.

[37] Song JQ, Bradeen JM, Naess SK, Raasch JA, Wielgus SM, Haberlach GT, et al Gene RB cloned from Solanum bulbocastanum confers broad spectrum resistance to potato late blight. P Natl Acad Sci USA. 2003;100(16):9128–33. 10.1073/pnas.1533501100 WOS:000184620000008. 12872003 PMC170883

[38] Jones JD, Dangl JL. The plant immune system. Nature. 2006;444(7117):323–9. 10.1038/nature05286 . 17108957

[39] Jallow MFA, Dugassa-Gobena D, Vidal S. Influence of an endophytic fungus on host plant selection by a polyphagous moth via volatile spectrum changes. Arthropod-Plant Inte. 2008;2(1):53–62. 10.1007/s11829-008-9033-8 WOS:000260961100006.

[40] Friedli J, Bacher S. Mutualistic interaction between a weevil and a rust fungus, two parasites of the weed Cirsium arvense. Oecologia. 2001;129(4):571–6. WOS:000172685100011. 10.1007/s004420100763 24577697

[41] Kruess A. Indirect interaction between a fungal plant pathogen and a herbivorous beetle of the weed Cirsium arvense. Oecologia. 2002;130(4):563–9. 10.1007/s00442-001-0829-9 28547258

[42] Rostás M, Hilker M. Asymmetric plant-mediated cross-effects between a herbivorous insect and a phytopathogenic fungus. Agricultural and Forest Entomology. 2002;4(3):223–31. 10.1046/j.1461-9563.2002.00147.x

[43] Dicke M, Baldwin IT. The evolutionary context for herbivore-induced plant volatiles: beyond the 'cry for help'. Trends in plant science. 2010;15(3):167–75. 10.1016/j.tplants.2009.12.002 WOS:000276519800007. 20047849

[44] Stout MJ, Fidantsef AL, Duffey SS, Bostock RM. Signal interactions in pathogen and insect attack: systemic plant-mediated interactions between pathogens and herbivores of the tomato, Lycopersicon esculentum. Physiological and Molecular Plant Pathology. 1999;54(3–4):115–30. 10.1006/pmpp.1998.0193 WOS:000079931500005.

[45] Cardoza YJ, Lait CG, Schmelz EA, Huang J, Tumlinson JH. Fungus-Induced Biochemical Changes in Peanut Plants and Their Effect on Development of Beet Armyworm, Spodoptera Exigua Hubner (Lepidoptera: Noctuidae) Larvae. Environmental Entomology. 2003;32(1):220–8. 10.1603/0046-225X-32.1.220

[46] D'Alessandro M, Erb M, Ton J, Brandenburg A, Karlen D, Zopfi J, et al Volatiles produced by soil-borne endophytic bacteria increase plant pathogen resistance and affect tritrophic interactions. Plant, cell & environment. 2014;37(4):813–26. 10.1111/pce.12220 . 24127750 PMC4194311

[47] Simon M, Hilker M. Herbivores and pathogens on willow: do they affect each other? Agricultural and Forest Entomology. 2003;5(4):275–84. 10.1046/j.1461-9563.2003.00189.x

[48] Gripenberg S, Mayhew PJ, Parnell M, Roslin T. A meta-analysis of preference-performance relationships in phytophagous insects. Ecology letters. 2010;13(3):383–93. 10.1111/j.1461-0248.2009.01433.x . 20100245

[49] Rao MS, Manimanjari D, Vanaja M, Rao CAR, Srinivas K, Rao VUM, et al Impact of elevated CO2 on tobacco caterpillar, Spodoptera litura on peanut, Arachis hypogea. J Insect Sci. 2012;12(103):1–10. WOS:000307928300001. 10.1673/031.012.10301 23437971 PMC3605029

[50] Mann RS, Ali JG, Hermann SL, Tiwari S, Pelz-Stelinski KS, Alborn HT, et al Induced release of a plant-defense volatile 'deceptively' attracts insect vectors to plants infected with a bacterial pathogen. PLoS pathogens. 2012;8(3):e1002610 10.1371/journal.ppat.1002610 22457628 PMC3310815

[51] Bradeen JM, Iorizzo M, Mollov DS, Raasch J, Kramer LC, Millett BP, et al Higher Copy Numbers of the Potato RB Transgene Correspond to Enhanced Transcript and Late Blight Resistance Levels. Molecular Plant-Microbe Interactions. 2009;22(4):437–46. 10.1094/Mpmi-22-4-0437 WOS:000264003600007. 19271958

