# Supplementary material for: Inoculation of Transgenic Resistant Potato by Phytophthora infestans Affects Host Plant Choice of a Generalist Moth
Source: PLoS One. 2015 Jun 8;10(6):e0129815. doi: 10.1371/journal.pone.0129815 (PMC4459979; doi:10.1371/journal.pone.0129815)
Supplement: S1 Fig — Expression anlaysis of the Rpi-blb1 in clone A01-22 plants, at different time points after inoculation, was quantified using qPCR. Error bars indicate standard deviation of the expression in relation to the EF1 internal control. (DOCX) [file pone.0129815.s001.docx]

**S1 Fig. Mean expression of the *Rpi-blb1* gene in the A01-22 clone.** Expression analysis of the *Rpi-blb1* in clone A01-22 plants, at different time points after inoculation, was quantified using qPCR. Error bars indicate standard deviation of the expression in relation to the EF1 internal control.
